# Hepatopancreatic metabolomics shedding light on the mechanism underlying unsynchronized growth in giant freshwater prawn, *Macrobrachium rosenbergii*

**DOI:** 10.1371/journal.pone.0243778

**Published:** 2020-12-23

**Authors:** Jianping Jiang, Xiang Yuan, Guanghua Huang, Wen Shi, Xueming Yang, Qinyang Jiang, Yinhai Jia, Xiurong Yang, Hesheng Jiang

**Affiliations:** 1 Guangxi Engineering Technology Research Center of Chinese Medicinal Materials Stock Breeding, Guangxi Botanical Garden of Medicinal Plants, Nanning, Guangxi, China; 2 College of Animal Science and Technology, Guangxi University, Nanning, Guangxi, China; 3 State Key Laboratory for Conservation and Utilization of Subtropical Agro-bioresources, Guangxi University, Nanning, Guangxi, China; 4 Guangxi Academy of Fisheries Sciences, Nanning, Guangxi, China; 5 Animal Husbandry Research Institute of Guangxi Zhuang Autonomous Region, Nanning, Guangxi, China; Fisheries and Oceans Canada, CANADA

## Abstract

The giant freshwater prawn, *Macrobrachium rosenbergii (M*. *rosenbergii)* as an important freshwater aquaculture species with high commercial value, exhibited unsynchronized growth. However, the potentially metabolic mechanism remains unclear. In this study, we used liquid chromatography tandem mass spectrometry (LC-MS/MS) to investigate the hepatopancreatic metabolic profiles of twenty giant freshwater prawns between the fast-growing group and slow-growing group. In the metabolomics assay, we isolated 8,293 peaks in positive and negative iron mode. Subsequently, 44 significantly differential metabolites were identified. Functional pathway analysis revealed that these metabolites were significantly enriched in three key metabolic pathways. Further integrated analysis indicated that glycerophospholipid metabolism and aminoacyl-tRNA biosynthesis have significant impact on growth performance in *M*.*rosenbergii*. Our findings presented here demonstrated the critical metabolites and metabolic pathways involved in growth performance, moreover provided strong evidence for elucidating the potentially metabolic mechanism of the unsynchronized growth in *M*. *rosenbergii*.

## 1. Introduction

The giant freshwater prawn, *Macrobrachium rosenbergii (M*. *rosenbergii)* is one of commercial important species around the world due to the special characteristic of nutrition-rich, fast-growing and higher economic values. In China, its production was up to 133,300 tons in 2018, which potentially contributed the most to its global production [1]. Similar to numerous crustaceans, *M*. *rosenbergii* exhibited unsynchronized growth pattern: some individuals grow fast, otherwise some are slowly growing. Notably, difference in growth rate was a crucial factor significantly affected yields of giant freshwater prawns. Over the past decades, large progresses have been made to understand the various internal and external factors, as well as the genetic factors, those influence individual growth variability in *M*. *rosenbergii* [2–4]. However, little is known regarding the metabolic mechanisms of unsynchronized growth.

Metabolomics as an analytical approach was applied to detect the low-molecular-weight metabolites [5]. While this method provides a glimpse of metabolic profiles, biomarkers and metabolic mechanism linked with human diseases [6,7], economic traits of plants [8] and domestic animals [9]. Also, metabolomics has been widely used in toxicity [10,11], sex differentiation [12], cold stress [13], flesh quality [14] and adaptation [15], moreover, it was widely utilized to growth performance [16,17] in aquaculture species. Otherwise, limited researches of metabolomics were published in *M*. *rosenbergii*. Bose et al. conducted untargeted metabolomics of the antennal gland (AnG), and identified several metabolites and biosynthetic pathway implicated in endogenous and exogenous transport [18]. Dong et al. performed muscle metabolomics of *M*. *rosenbergii* by treating with different concentration of ammonia-N (0, 0.108, 0.324, or 0.54 mg L−1) for 20 days. Subsequently, a list of metabolomics pathways related to lipid, carbohydrate, and protein metabolism were identified, which was contributed to illustrate the mechanisms underlying the effects of ammonia stress in *M*. *rosenbergii* [11]. Until now, the metabolic profiles and metabolites regarding the unsynchronized growth of *M*. *rosenbergii* was scarce.

Therefore, the objective of the present study was used the liquid chromatography tandem mass spectrometry (LC-MS/MS) to investigate the metabolic profiles of *M*. *rosenbergii*, and further to detect the differential metabolites between the fast-growing and slow-growing groups. As expected, the results we obtained could provide a clue for illustrating the metabolic mechanism to understand the unsynchronized growth of *M*. *rosenbergii*.

## 2. Materials and methods

### 2.1. Ethics statement

All procedures were in compliance with the institutional guidelines and under a protocol approved by the Animal Experimental Ethical Inspection Form of Guangxi Botanical Garden of Medicinal Plants.

### 2.2. Animals

The prawn population was established in the national *Macrobrachium rosenbergii* seed multiplication farm, Nanning, Guangxi, China. A total of 20 mating pairs (female: male = 20:20) were constructed to produce the progeny stock. In August 2019, family production was finished. The subsequent procedure for hatching and rearing was according to Hung et al. [19]. Totally, 200 juveniles from each family were randomly selected and reared into grow-out ponds. Finally, each of 10 prawns from one family with extremely growth performance were chosen as fast-growing and slow-growing individuals (Table 1).

**Table 1:** Growth performance of 20 prawns between fast-growing and slow-growing groups.

	Fast-growing group	Slow-growing group
Body weight (g)	10.33±0.93^A^	4.87±0.38^B^
Body length (cm)	6.4±0.28^A^	5.07±0.15^B^

### 2.3. Sample preparation

The hepatopancreas of each sample from fast-growing and slow-growing groups was immediately dissected, and then stored in liquid nitrogen. Sample preparation for LC-MS/MS analysis was conducted as previously described by Want et al. [20]. Briefly, samples of 100 mg were mixed with 1 mL chilled extraction liquid (methanol: water = 4:1, vol: vol) containing 2-Chloro-L-phenylalanine (Shanghai Hanhong Scientific Co.,Ltd.) as internal standard, vortexed for 30 s, and homogenized to extract the compounds from the hepatopancreas. Then, the homogenate was further ultrasonically treated in ice bath for 3 min, and deproteinized through centrifugation at 4°C (12,000 rpm, 10 min). The supernatant was subsequently transferred into a new microcentrifuge tube and lyophilized. The dried samples were reconstructed with chilled methanol/water (4:1, v: v) for further process.

### 2.4. LC–MS/MS analysis

Briefly, the LC-MS/MS experiments were performed on the Dionex UltiMate 3000 Uhplc system coupled with Q Exactive mass spectrometer (Thermo Fisher Scientific, CA, USA) operating in data-dependent acquisition (DDA) mode. Samples were injected onto a Hypersil GOLD HPLC column (50×2.1 mm, 1.9 μm). The mobile phase consisted of a gradient system of (A) 10 mM ammonium formate in water and (B) 10 mM ammonium formate in methanol: 0–2 min, 5% B; 2–5 min, 5–30% B; 5–19 min, 30–99% B; 19–22 min, 99% B; and 22.1–25 min, 5% B.

Compound ionization was conducted as the following parameters: Q-Exactive mass spectrometer was operated in positive/negative polarity mode with spray voltage 3.5 kV/3.2 kV, capillary temperature of 320°C, sheath gas flow rate 30 psi and aux gas flow rate 10 arb. Samples were analyzed using liquid chromatography-high resolution mass spectrometry (LC-HRMS) in full scan + data dependent MS2 mode with a scan range from 100–1000 m/z at a resolution of 70,000, followed by data dependent MS/MS (dd-MS/MS) with a normalized collision energy of 30 and at a resolution of 17,500. To avoid instrument drift, fourteen quality control (QC) samples were preprocessed as the samples for data quality assessment.

### 2.5. Data analysis

Data processing including peak alignment, retention time correction, and peak area extraction was conducted by commercially available software, Compound Discoverer v. 3.0 (Thermo Fisher Scientific, CA, USA). The identified metabolites were searched against mzCloud and ChemSpider database. For multivariate statistical analysis, principal component analysis (PCA) and orthogonal partial least-squares discriminant analysis (OPLS–DA) were performed to detect the metabolic variations between the two experimental groups through the SIMCA-P v.14.1 software (Umetrics, Umeå, Sweden) after pareto (Par) scaling. The quality of OPLS-DA model was assessed based on the cumulative parameters R^2^X, R^2^Y, and Q^2^ in cross-validation, and applying a permutation test with 200 permutations.

Significantly differential metabolites of the pairwise comparisons were identified with VIP score > 1 obtained from OPLS-DA model and *p* < 0.05 from Student’s t test. Hierarchical cluster analysis (HCA) was performed via TBtools software v.1.046 [21]. Afterward, pathway enrichment analysis of identified metabolites was carried out through MetaboAnalyst v.4.0 software [22] (https://www.metaboanalyst.ca/).

## 3. Results

### 3.1. Overall data set and metabolic profiles

In total, 5,589 and 2,704 peaks were detected in positive and negative ion modes, respectively. Subsequently, after further quality control filtering, 1,254 and 222 peaks were retained for parallel analyses.

To characterize the variations in the metabolic profiles of *M*.*rosenbergii* between the fast-growing group and slow-growing group, PCA and OPLS-DA were conducted.

**Fig 1:**
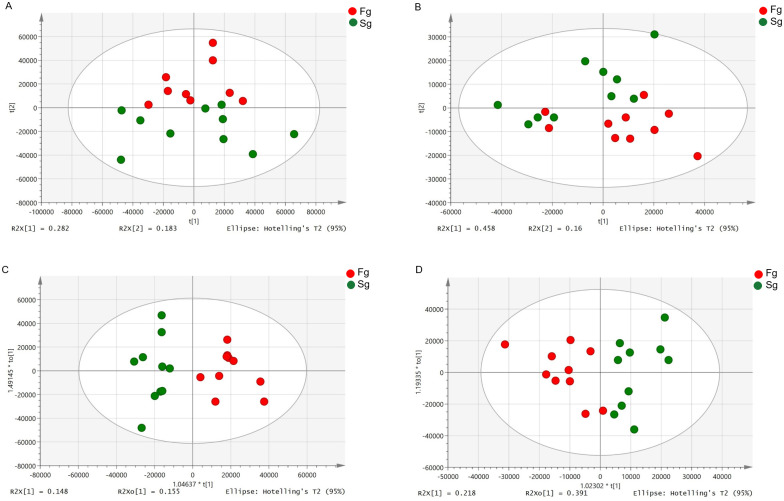
PCA (A and B, in positive and negative modes, respectively) and OPLS-DA (C and D, positive and negative modes, respectively) scores plots based on LC-MS/MS data of hepatopancreas samples from Fg (red) and Sg (green).

**Fig 2:**
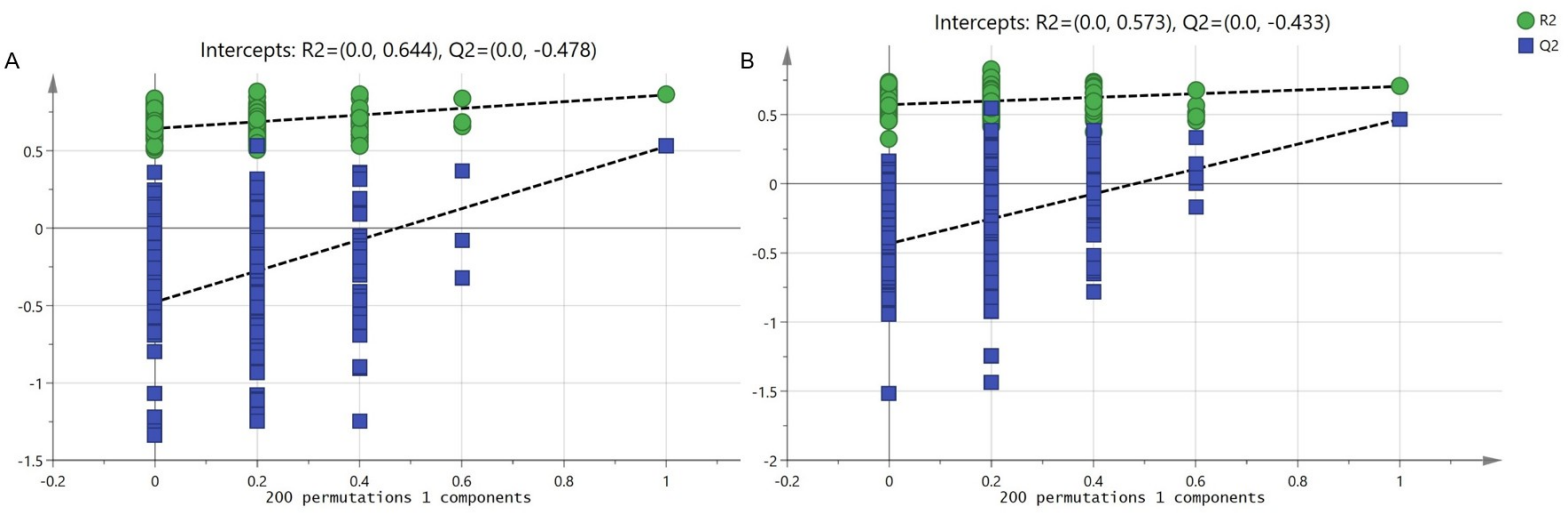
OPLS-DA permutation test for positive (A) and negative ion mode (B).

As shown in Fig 1A and 1B, apparent separation was observed of the 20 prawns between fast-growing and slow-growing groups. The percentage of explained value in the metabolomics analysis of PC1 and PC2 was 28.2% and 18.3% (positive ion mode), 45.8% and 16.0% (negative ion mode), respectively. Subsequently, a further examination based on the OPLS-DA score plot showed a clear separation between the two groups. In the positive (negative) ion mode, the parameters considered for classification from the software were R^2^X (cum) = 0.303, R^2^Y (cum) = 0.860, Q^2^ (cum) = 0.528 (R^2^X (cum) = 0.608, R^2^Y (cum) = 0.704, Q^2^ (cum) = 0.467) (Fig 1C and 1D). Subsequently, model cross-validation through permutation tests (200 times) generated intercepts of R^2^ and Q^2^ (positive ion mode, 0.644 and -0.478; negative ion mode, 0.573 and -0.433, respectively) (Fig 2). Herein, all of which confirmed that the OPLS-DA model was stable and not over-fitted. Taken together, multivariate analyses (PCA and OPLS-DA) demonstrated a clear and significant separation of the fast-growing group versus slow-growing group.

### 3.2. Significantly differential metabolites

**Fig 3:**
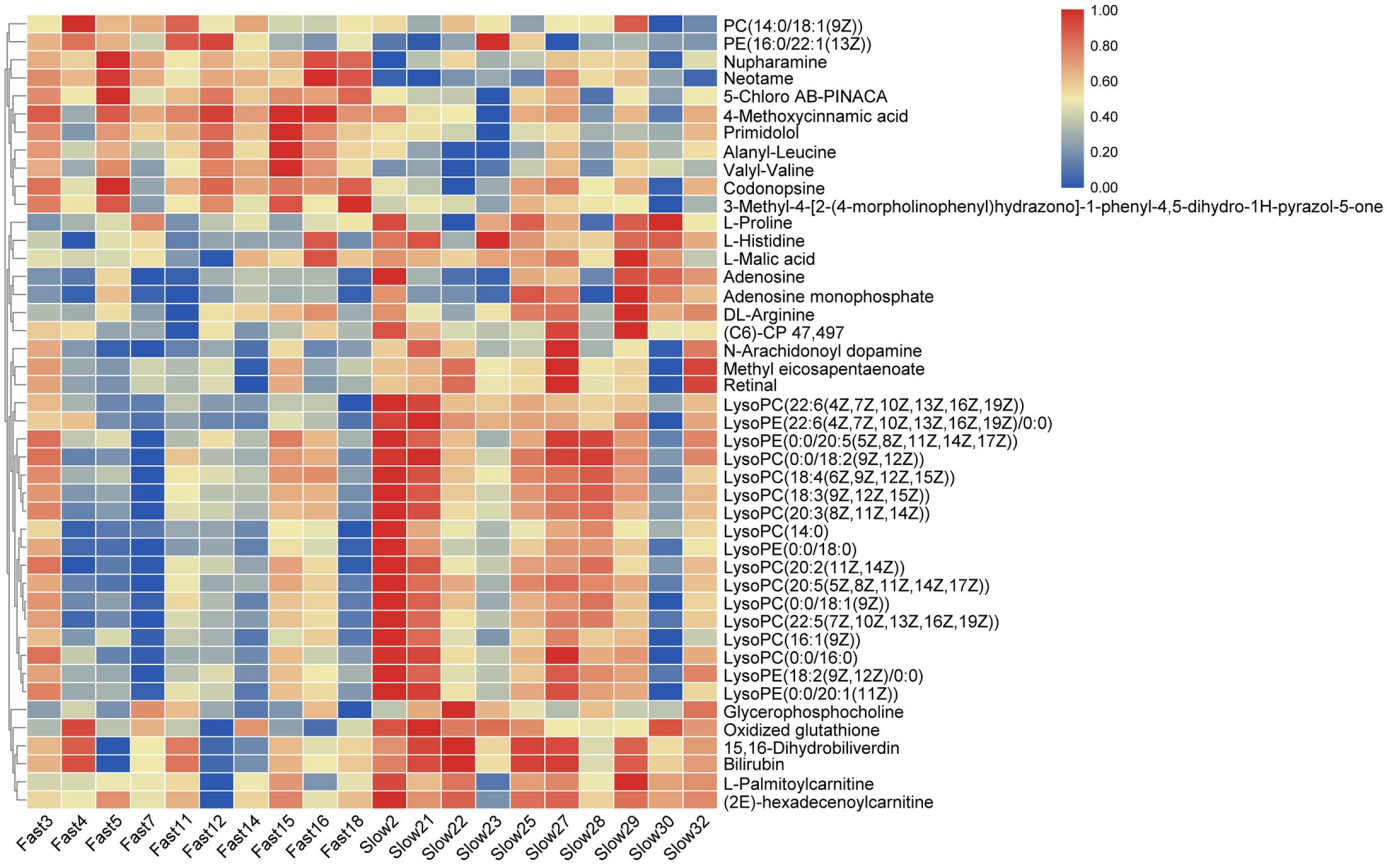
Heatmap of significantly differential metabolites between the fast-growing and slow-growing groups.

On the basis of the OPLS-DA results, a total of 44 (36 in positive ion mode and 8 in negative ion mode) significantly differential metabolites (SDMs) were identified (VIP > 1, *p* < 0.05) between the fast-growing and slow-growing groups (Table 2), and MS/MS spectrums of seven representative SDMs were listed in additional file 1. Among the 44 SDMs, 11 and 33 metabolites were significantly up-regulated and down-regulated compared with those in slow-growing group. Hierarchical clustering analysis also indicated that each type of the two groups exhibited a distinct metabolic pattern (Fig 3).

**Table 2:** Significantly differential metabolites for 20 prawns between fast-growing and slow-growing groups.

**Significantly differential metabolites identified in positive ion mode**						
**No.**	**Metabolites**	**MW(Da)**	**RT(min)**	**Log**_**2**_**FC**	***P*-value**	**VIP**
1	LysoPC(0:0/18:2(9Z,12Z))	519.332	17.303	-1.33	0.0014	13.8337
2	LysoPC(0:0/18:1(9Z))	521.348	17.922	-0.78	0.0144	9.9551
3	LysoPC(20:5(5Z,8Z,11Z,14Z,17Z))	541.316	16.71	-1.81	0.0010	7.7474
4	LysoPC(0:0/16:0)	495.332	17.665	-0.99	0.0351	4.7933
5	LysoPC(18:3(9Z,12Z,15Z))	517.316	16.704	-1.58	0.0012	4.6793
6	PC(14:0/18:1(9Z))	731.545	21.13	0.26	0.0086	3.9071
7	LysoPC(22:6(4Z,7Z,10Z,13Z,16Z,19Z))	567.331	17.471	-1.12	0.0004	3.7332
8	Methyl eicosapentaenoate	316.240	18.56	-0.66	0.0479	3.4406
9	Glycerophosphocholine	257.102	0.458	-0.78	0.0324	3.3007
10	_L_-Arginine	174.112	0.487	-0.53	0.0250	3.2579
11	LysoPC(16:1(9Z))	493.316	16.885	-0.64	0.0353	3.1972
12	15,16-Dihydrobiliverdin	584.263	14.911	-2.56	0.0022	2.9967
13	4-Methoxycinnamic acid	178.063	4.534	0.23	0.0436	2.7188
14	Retinal	284.214	18.555	-0.67	0.0390	2.2772
15	LysoPC(22:5(7Z,10Z,13Z,16Z,19Z))	569.347	17.58	-1.33	0.0020	2.2174
16	PE(16:0/22:1(13Z))	773.592	21.839	0.41	0.0480	2.1821
17	LysoPC(14:0)	467.301	16.539	-1.28	0.0002	2.0772
18	LysoPE(0:0/20:5(5Z,8Z,11Z,14Z,17Z))	499.269	16.673	-1.22	0.0138	2.0684
19	Adenosine	267.097	2.057	-1.94	0.0235	2.0605
20	LysoPC(20:2(11Z,14Z))	547.363	18.217	-1.47	0.0016	1.9187
21	_L_-Proline	115.063	0.506	-0.54	0.0468	1.7536
22	_L_-Palmitoylcarnitine	399.334	17.702	-0.79	0.0435	1.6945
23	Nupharamine	251.188	13.506	1.14	0.0053	1.6195
24	Alanyl-Leucine	202.132	4.516	0.38	0.0283	1.6171
25	LysoPC(20:3(8Z,11Z,14Z))	545.347	17.707	-1.42	0.0018	1.5545
26	LysoPE(18:2(9Z,12Z)/0:0)	477.285	17.238	-1.17	0.0242	1.4848
27	LysoPE(0:0/20:1(11Z))	507.332	17.434	-0.94	0.0306	1.2884
28	LysoPE(0:0/18:0)	481.316	17.138	-1.32	0.0021	1.2403
29	LysoPE(22:6(4Z,7Z,10Z,13Z,16Z,19Z)/0:0)	525.285	17.397	-1.29	0.0095	1.2357
30	Primidolol	333.169	6.939	0.45	0.0319	1.2201
31	trans-Hexadec-2-enoyl carnitine	397.319	16.896	-0.88	0.0491	1.1108
32	Codonopsine	267.147	9.468	1.52	0.0193	1.0629
33	Oxidized glutathione	612.151	0.434	-0.82	0.0189	1.0456
34	Valyl-Valine	216.147	5.706	0.27	0.0229	1.0404
35	Adenosine monophosphate	347.063	0.629	-3.3	0.0446	1.0208
36	LysoPC(18:4(6Z,9Z,12Z,15Z))	515.301	16.117	-1.78	0.0048	1.0113
**Significantly differential metabolites identified in negative ion mode**						
**No.**	**Metabolites**	**MW(Da)**	**RT(min)**	**Log**_**2**_ **FC**	***P*-value**	**VIP**
1	Neotame	378.217	14.489	1.28	0.0015	5.4073
2	CP 47,497-C6-Homolog	304.239	17.776	-0.11	0.0301	2.1848
3	N-Arachidonoyl dopamine	439.304	18.267	-1.05	0.0172	1.7745
4	Bilirubin	584.261	14.917	-2.29	0.0022	1.7004
5	_L_-Histidine	155.068	0.505	-0.41	0.0077	1.6767
6	5-Chloro AB-PINACA	364.165	10.048	0.93	0.0107	1.2014
7	3-Methyl-4-[2-(4-morpholinophenyl)hydrazono]-1-phenyl-4,5-dihydro-1H-pyrazol-5-one	363.170	10.633	0.49	0.0161	1.1153
8	L-Malic acid	134.020	0.455	-0.36	0.0471	1.0751

### 3.3. Metabolic KEGG pathway analysis

**Fig 4:**
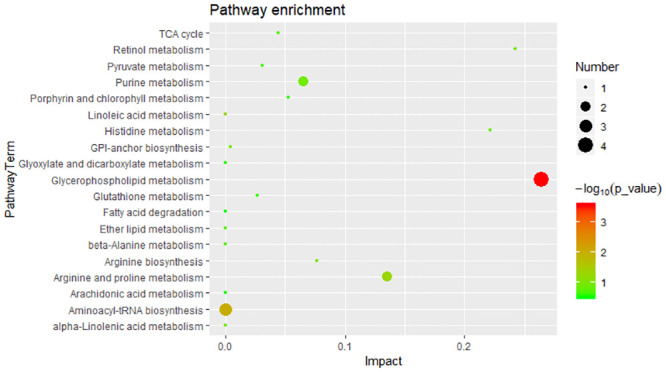
Significant metabolic pathways for 44 SDMs.

To explore potentially metabolic pathways affected by different growth performances, pathway analysis of 44 SDMs were performed by MetaboAnalyst 4.0. Functional analysis revealed that the metabolites those significantly difference were involved in glycerophospholipid metabolism, aminoacyl-tRNA biosynthesis and linoleic acid metabolism (Fig 4). The putative compounds hit LysoPC (20:5(5Z,8Z,11Z,14Z,17Z)) (LysoPC), PC (14:0/18:1(9Z)) (PC), Glycerophosphocholine (GPC), PE (16:0/22:1(13Z)) (PE), _L_-histidine, _L_-arginine and _L_-proline.

## 4. Discussion

In the past decades, unsynchronized growth of *M*. *rosenbergii* have caused severe productive and economic losses, while the potentially metabolic mechanism behind the phenomenon remains unclear. Hepatopancreas as the major organ, is implicated in carbohydrate and energy metabolism, protein and lipid synthesis [23]. Moreover, it plays an important role in the synthesis and secretion of digestive enzymes, nutrient absorption, digestion, reserve storage and mobilization [24]. Accumulating evidence have shown that hepatopancreas has significant impact on crustacean growth [25]. Thus, in the present study, we performed hepatopancreatic metobolomics of giant freshwater prawns with different growth performance between the fast-growing group and slow-growing group based on the LC-MS/MS. To our knowledge, this study was the first investigation to identify the key metabolites and pathways implicated in growth performance, which will provide novel insights into understanding of metabolic mechanism underlying the unsynchronized growth in *M*. *rosenbergii*.

**Fig 5:**
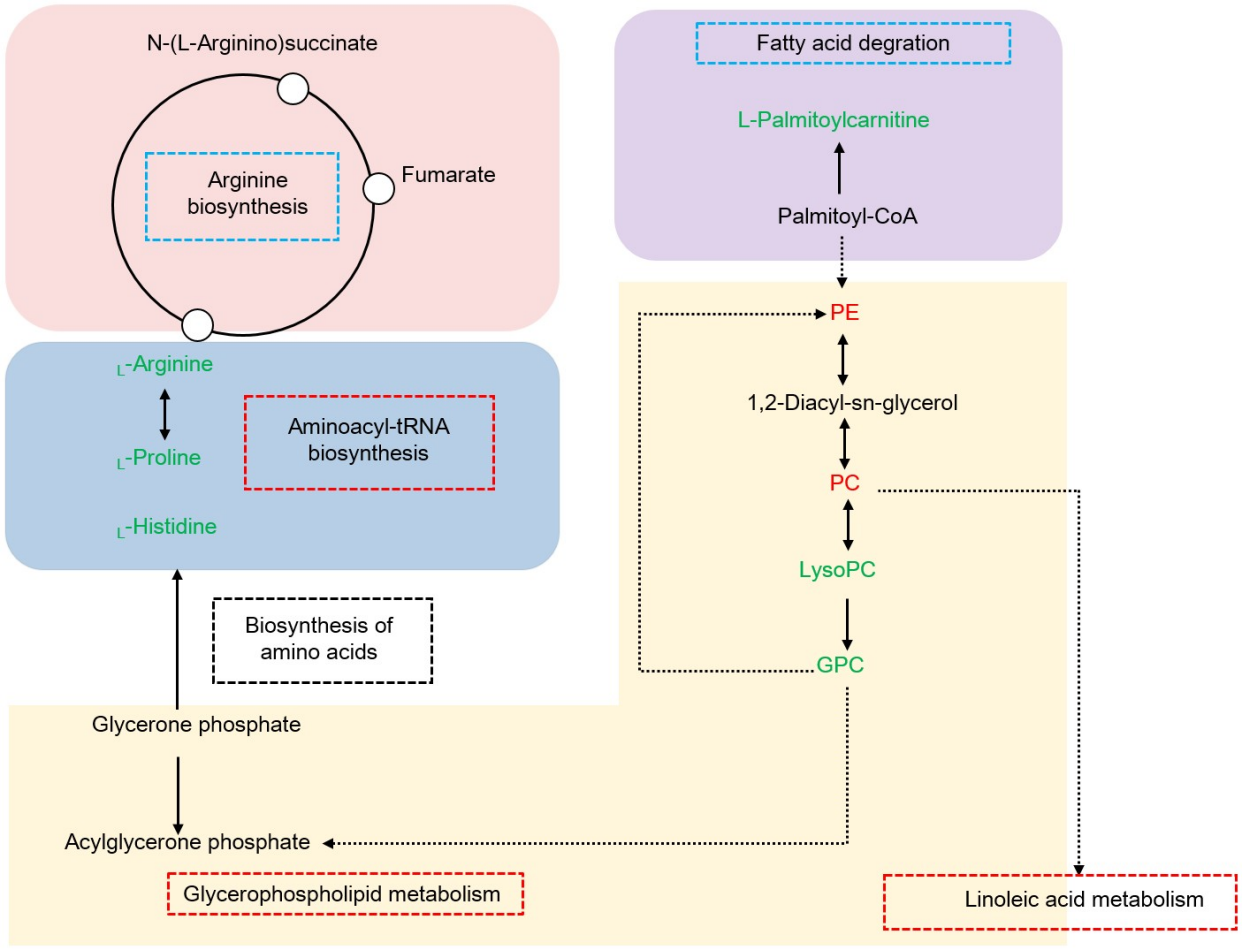
Hypothesized pathway of the different growth performances of *M*. *rosenbergii*. Up-regulated and down-regulated metabolites are shown in red or green letters. Three significant pathways are shown in red boxes, two pathways not significantly enriched are shown in blue boxes. Abbreviations: PE, PE (16:0/22:1(13Z)); PC, PC (14:0/18:1(9Z)); LysoPC, LysoPC (20:5(5Z,8Z,11Z,14Z,17Z)); GPC, Glycerophosphocholine.

Of note, combining our data and KEGG database, a comprehensive scheme that controlling growth performance of *M*. *rosenbergii* is proposed, as shown in Fig 5. We speculated that in the hepatopancreas, glycerophospholipid metabolism might affect the physiological functions of cells and membranes, moreover provide energy for aminoacyl-tRNA biosynthesis and amino acid biosynthesis via fatty acid degradation and oxidation in response of different growth performance in *M*. *rosenbergii*.

### 4.1. Glycerophospholipid metabolism and linoleic acid metabolism

Amongst, the elements of glycerophospholipid metabolism, including LysoPC (20:5(5Z,8Z,11Z,14Z,17Z)), PC (14:0/18:1(9Z)), Glycerophosphocholine and PE (16:0/22:1(13Z)) were significantly altered between the two groups. It has been reported that PC and PE are the main lipid constituents of cell membranes, which play critical roles in functioning well of cells [26]. Decreasing the PC content affected the integrity of liver cells and mitochondrial membrane, thus directly leading to proliferation, differentiation and apoptosis [27]. LysoPC is the main component of low density lipoprotein. Generally, the content of LysoPC was low occurred in cells or tissues, high concentrations could damage the membrane system of cells [28].

Glycerophosphocholine is produced by lysoPC [29], which was response for cell viability and motility [30]. Some studies have proposed that higher content of GPC acted as an indicator of cancer progression [31]. Notably, PC was involved in linoleic acid metabolism. Interestingly, in recent prior studies, researchers have observed that glycerophospholipid metabolism and linoleic acid metabolism have closed relationship with energy metabolism via β-oxidation [32,33]. The present study investigated that PC and PE, LysoPC and GPC were shown down-regulated and up-regulated in fast-growing group, respectively. Overall, the data could interpret that high concentration of PC and PE, low concentration of LysoPC and GPC was essential for different growth performance.

### 4.2. Aminoacyl-tRNA biosynthesis

For the aminoacyl-tRNA biosynthesis, previous researches have observed that it is an important metabolic pathway before amino acid biosynthesis [34]. In this study, three amino acids including _L_-histidine, _L_-arginine, _L_-proline those identified in the metabolic pathway showed down-regulated in fast-growing group.

Zhao et al. demonstrated that dietary histidine level affected the growth performance, body composition of juvenile Jian carp [35]. Similarly, Zehra et al. also confirmed that dietary histidine level has positive effects on the growth performance, protein deposition and carcass composition [36]. _L_-Arginine (Arg) is synthesized from glutamine, glutamate and proline. It has demonstrated that the concentration of Arg in hepatocytes was very low [37]. It has been found that dietary arginine contributed to the growth performance, immunity and health status of broiler chicks [38]. _L_-Proline as an essential precursor for the synthesis of proteins most publications have paid attention to proline on plants [39,40]. It was indicated that proline displays remarkable role on plant growth and development under non-stress or stress conditions, but the role of proline on growth was varied [41].

Meanwhile, the three amino acids have favorable roles in formation of peptide chains. Moreover, Wang et al. proposed that aminoacyl-tRNA biosynthesis was significantly associated with the growth of D. similis [34], which was in agreement with previous finding of Yebra et al. [42]. All in all, high contents of _L_-histidine, _L_-arginine, _L_-proline might disrupt the homeostasis of hepatopancreas, and perturb the transport process to the intestine or plasma.

## 5. Conclusions

In summary, we investigated the metabolic profiles of hepatopancreas between the fast-growing group and slow-growing group in *M*. *rosenbergii* based on the LC-MS/MS, and identified 44 significantly differential metabolites. Integrated analysis of key metabolic pathways showed that glycerophospholipid metabolism and aminoacyl-tRNA biosynthesis played crucial role in response to unsynchronized growth. Notably, seven metabolites, consist of LysoPC(20:5(5Z,8Z,11Z,14Z,17Z)), PC(14:0/18:1(9Z)), Glycerophosphocholine, PE(16:0/22:1(13Z)), _L_-histidine, _L_-arginine and _L_-proline, were strongly correlated with growth performance. The results obtained in our study demonstrated the critical pathways and metabolites to decipher the potential metabolic mechanism of the unsynchronized growth in *M*. *rosenbergii*.

## Supporting information

S1 Fig MS/MS spectrum of PC(14:0/18:1(9Z)). (DOCX)

S2 Fig MS/MS spectrum of LysoPC(20:5(5Z,8Z,11Z,14Z,17Z)). (DOCX)

S3 Fig MS/MS spectrum of Glycerylphosphorylcholine. (DOCX)

S4 Fig MS/MS spectrum of PE(16:0/22:1(13Z)). (DOCX)

S5 Fig MS/MS spectrum of _L_-Histidine. (DOCX)

S6 Fig MS/MS spectrum of _L_-Arginine. (DOCX)

S7 Fig MS/MS spectrum of _L_-Proline. (DOCX)
