# Supplementary material for: Hepatopancreatic metabolomics shedding light on the mechanism underlying unsynchronized growth in giant freshwater prawn, Macrobrachium rosenbergii
Source: PLoS One. 2020 Dec 23;15(12):e0243778. doi: 10.1371/journal.pone.0243778 (PMC7757812; doi:10.1371/journal.pone.0243778)
Supplement: S4 Fig — (DOCX) [file pone.0243778.s004.docx]

Fig.4. MS/MS spectrum of PE(16:0/22:1(13Z))
